# Reviewer acknowledgement 2013

**DOI:** 10.1186/1759-8753-4-4

**Published:** 2013-02-25

**Authors:** Nancy L Craig, Thomas H Eikbush, Daniel F Voytas

**Affiliations:** 1 Johns Hopkins School of Medicine, 725 N. Wolfe Street, 502 PCTB, Baltimore, MD 21205, USA; 2 Department of Biology, University of Rochester, River Campus, Box 270211, Rochester, NY 14627-0211, USA; 3 Microbial and Plant Genomics Institute, University of Minnesota, 340 Cargill Building, 1500 Gortner Avenue, Saint Paul, MN 55108, USA

## Abstract

**Contributing reviewers:**

The editors of Mobile DNA would like to thank all our reviewers who have contributed to the journal in volume 3 (2012).

## 

Peter Arensburger

United States of America

Irina Arkhipova

United States of America

Frederick Arnaud

France

Joshua Baller

United States of America

Jef Boeke

United States of America

Stephane Boissinot

United States of America

Guillaume Bourque

Singapore

Richard Calendar

United States of America

Josep Casacuberta

Spain

Ronald Chalmers

Afghanistan

Shawn Christensen

United States of America

Joan Curcio

United States of America

Thomas Eikbush

United States of America

Astrid Engel

United States of America

Malcolm Fraser

United States of America

Anthony Furano

United States of America

Xiang Gao

United States of America

David Garfinkel

United States of America

Vera Gorbunova

United States of America

Heidrun Gundlach

Germany

Graham Hatfull

United States of America

Zoltan Ivics

Germany

I. King Jordan

United States of America

I. King Jordan

United States of America

Ruslan Kalendar

Finland

Haig Kazazian

United States of America

Kenji Kojima

United States of America

Howard Laten

United States of America

Emmanuelle Lerat

France

Jiri Macas

Czech Republic

Dixie Mager

Canada

Sandy Martin

United States of America

Jens Mayer

Germany

Didier Mazel

France

John Moran

United States of America

Ellen Pritham

United States of America

David Ray

United States of America

Phoebe Rice

United States of America

Sylvie Rimsky

France

Alfredo Ruiz

Spain

Suzanne Sandmeyer

United States of America

Sarah Schaack

United States of America

Gerald Schumann

Germany

David Symer

United States of America

Sebastien Tempel

France

Christophe Terzian

France

Chantal Vaury

France

Alexander Vershinin

Russia

Jean-Nicolas Volff

Germany

Daniel Voytas

United States of America

Holly Wichman

United States of America

David Witherspoon

United States of America

Jinchuan Xing

United States of America

Steven Zimmerly

Canada

